# CEP44 and CCDC15 label the spermatozoa proximal and atypical distal centrioles

**DOI:** 10.17912/micropub.biology.001393

**Published:** 2025-01-07

**Authors:** Luke Achinger, Briggs Hehl, Jason Suh, Samantha B. Schon, Nagalakshmi Nadiminty, Tariq A. Shah, Puneet Sindhwani, Tomer Avidor-Reiss

**Affiliations:** 1 University of Toledo, Toledo, Ohio, United States; 2 University of Michigan–Ann Arbor, Ann Arbor, Michigan, United States; 3 The University of Toledo

## Abstract

The centrosome is a conserved characteristic of eukaryotic and human cells but is highly specialized in reproductive cells. The spermatozoan centrosome includes a slightly modified proximal centriole, an atypical distal centriole, and specialized pericentriolar material, including striated columns and capitellum. We investigated the localization of canonical centriolar proteins CEP44 and CCDC15 in human spermatozoa. We found that CEP44 localizes mainly at the proximal centriole and distal centriole bases relative to centrin. CCDC15 colocalizes with centrin in both the proximal centriole and distal centriole. These findings further our understanding of the spermatozoan centrosome composition.

**Figure 1. CEP44 and CCDC15 label the spermatozoa proximal and atypical distal centrioles: CEP44 adjacent to centrin, and CCDC15 colocalizes with centrin.**
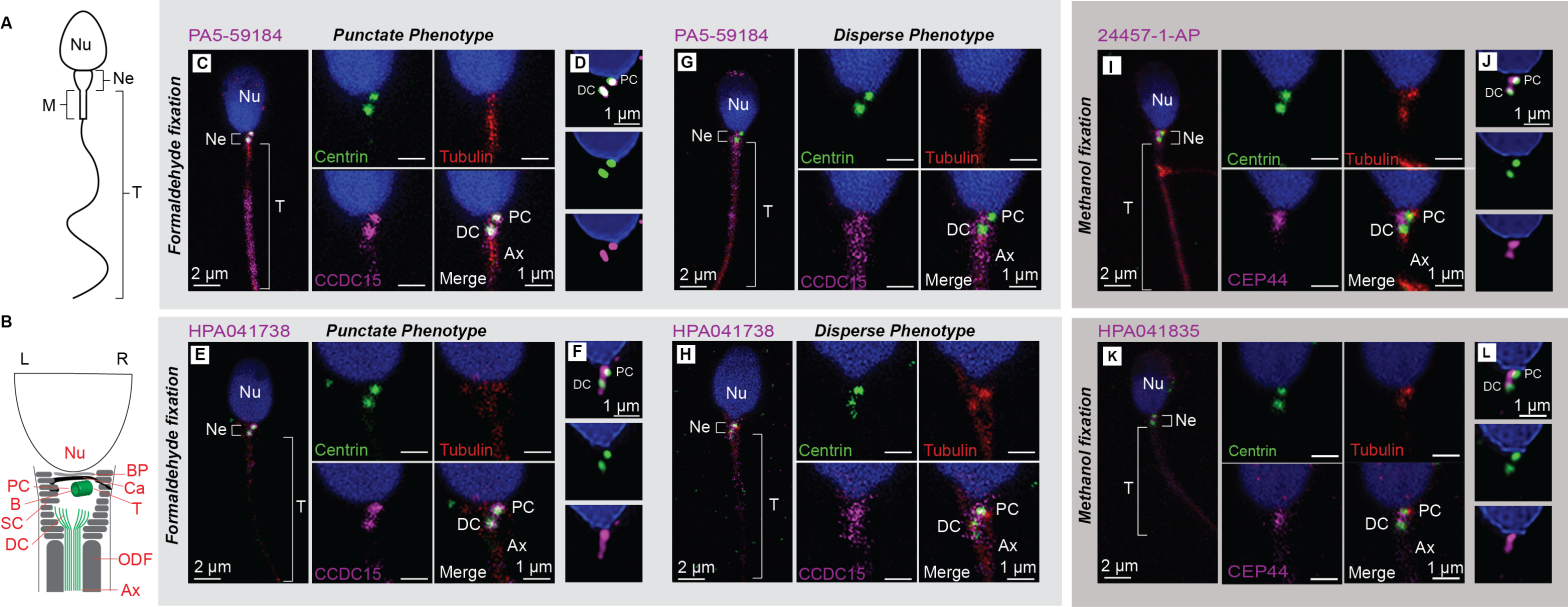
**A)** Diagram depicting the spermatozoa’s main parts, including the nucleus (Nu), neck (Ne), midpiece (M), and tail (T). **B** ) A zoom-in on the sperm neck substructures. Spermatozoa are asymmetric in their rostral-caudal and left-right axes. Therefore, it is depicted with the head up and proximal centriole on the right (R) for convenient comparison. The spermatozoan neck lies just below the base of the nucleus (Nu). It is made up of a basal plate (BP), capitulum (Ca), proximal centriole (PC) with a medial base (B) and lateral tip (T), distal centriole (DC), outer dense fibers (ODF), striated columns (SC), and the beginning of the axoneme (Ax). **C-H)** CCDC15 localized at the proximal and distal centrioles, closer to the centriole base than centrin in the punctate phenotype (antibody PA5-59184 in **C-D** and antibody HPA041738 in **E-F** ). CCDC15 also has a dispersed phenotype around the proximal and distal centrioles ( **G, H** ). **I-L)** CEP44 co-localized with centrin at the proximal and distal centriole (antibody 24457-1AP in **I-J** , antibody HPA041835 in **K-L** ). Centrin (green) localizes to the proximal and distal centrioles, tubulin (red) labels the centrioles and axoneme, and DAPI (blue) labels the nucleus in the head. Panels C, E, G, H, I, and K are low-magnification confocal images on the left (scale bar 2 µm) and four high-magnification confocal images (scale bar 1 µm) from the same spermatozoa. HyVolution images **(D, F, J, and L)** from separate spermatozoa (scale bar 1 µm).

## Description

Centrioles are essential microtubule nucleation hubs in human somatic cells throughout the cell cycle. During cell division, centrioles form centrosomes that act as cytoplasmic microtubule-organization centers, and abnormalities in these structures are associated with morbidities, such as cancer (Fisk et al., 2019; Marteil et al., 2018; Nigg & Raff, 2009) . During interphase, the centrioles nucleate cilia, and abnormalities in these structures lead to various diseases called ciliopathies (Nigg & Raff, 2009) . A cell has two centrioles during the early interphase, and four centrioles after the S phase of the cell cycle. Canonical centrioles consist of nine microtubule triplets assembled into a barrel-like structure (Hirono, 2014) . Centrioles are often found in pairs composed of a mother and daughter centriole, differentiated by the proteinaceous distal appendages around the mother centriole tip and proteinaceous cartwheel in the lumen of the daughter centriole base (Hung et al., 2016) . In a centrosome, the centrioles are surrounded by a pericentriolar matrix that nucleates and anchors cytoplasmic microtubules (Conduit et al., 2015) .

The spermatozoan centrosome is the main structure that links the sperm head and tail ( **Fig 1A** ), also known as the sperm neck or head-tail coupling apparatus (Wu et al., 2020) . Mutating sperm neck and centrosomal proteins such as CEP112, CEP135, and POC1B can produce acephalic sperm or abnormal tail morphology (Ge et al., 2024; Hua et al., 2023; Li et al., 2022; Sha et al., 2020; Sha et al., 2017; Shang et al., 2017; Wang et al., 2023; Wang et al., 2022; Zhu et al., 2018) . Like somatic centrosomes, the spermatozoan centrosome consists of two centrioles: the proximal and distal centrioles ( **Fig 1B** ). However, these centriole’s structure and composition are modified throughout spermiogenesis in a process referred to as centriole remodeling or centrosome reduction (Khire et al., 2016; Manandhar et al., 2000) . As a result, the distal centriole is dramatically altered, and its microtubules splay, rendering it elastic and permitting its left and right sides to slide to shape the sperm tail beating pattern (Khanal et al., 2021) . The proximal centriole is also mildly modified from its canonical composition and structure (Fishman et al., 2018) . The pericentriolar matrix becomes the striated columns, the central mechanical linker connecting the head and the tail (Manandhar & Schatten, 2000) .

Human spermatozoa express ~450 somatic centrosome proteins (Khanal et al., 2024) ; only a few have known localization patterns. The spermatozoan centrioles include canonical centriolar luminal proteins WDR90, FAM161A, Centrin-1/2, POC1B, POC5, CEP63, and CEP135, as well as other centriolar proteins such as P110, CPAP, and tubulin with acetylation and glutamination (Fishman et al., 2018; Khanal et al., 2021; Sha et al., 2017; Subbiah et al., 2024; Turner et al., 2022) . They also contain other proteins such as WDR62, Nek9, Potin, and Reptin (Amargant et al., 2021) . Because many canonical centriolar luminal proteins appear to be present in the sperm centrioles, this study examined the localization of two distinct canonical centriole luminal proteins: CCDC15, found within the inner lumen in somatic cells (Arslanhan et al., 2023) , and CEP44, found at the proximal lumen in somatic cells (Hossain et al., 2020) .

Centrin, a canonical centriole distal lumen protein (Le Guennec et al., 2020) , labels both the proximal and distal centrioles in human sperm (Fishman et al., 2018) . We used it to mark the location of the spermatozoa centrioles ( **Fig 1C-L** ). Usually, centrin labels two distinct neck points: one just adjacent to the DAPI-labeled nuclear base – the proximal centriole, and a second within 1 µm from the nucleus and just rostral to the tubulin-labeled axoneme – the distal centriole. Indeed, 84% of spermatozoa (123/147) imaged had centrin in both the proximal centriole and distal centriole, 6% (9/472) had only proximal centriole labeling, 1% (2/147) had only the distal centriole labeled, and 8% (12/147) were inconclusive. We noticed that, proportionally, in spermatozoa fixed in methanol, centrin labeling was an average of 38% more intense in the proximal centriole than the distal centriole (1.38±0.75-fold more intense, P=2E-5, nomber of sperm N=69, paired t-test). However, in spermatozoa fixed in formaldehyde, centrin labeled the PC and DC with equal intensity (P=0.96, N=36, paired t-test). This centrin intensity difference is possibly due to different immunoreactivity because of differences in antibody accessibility, centrin conformation, posttranslational modification, or interaction with other proteins.

Coiled-coil domain-containing protein 15, or CCDC15, is a protein that localizes to the central region of centrioles in the human retinal pigment epithelium (RPE1) cell line (Arslanhan et al., 2023) . This study also reported that CCDC15 colocalizes with inner scaffold proteins such as centrin. Like centrin, CCDC15 has been implicated in regulating the size and integrity of centrioles and the assembly of functional cilia. We visualized and labeled CCDC15 using two commercial antibodies, PA5-59184 (ThermoFisher Scientific) and HPA041738 (Sigma), using formaldehyde fixatives. These antibodies did not label the sperm’s neck consistently when using methanol as a fixative.

PA5-59184 is a rabbit polyclonal antibody raised against amino acids 291-370 of human CCDC15. PA5-59184 yielded two different labeling patterns in human spermatozoa: punctate pattern ( **Fig 1C-F** ) and dispersed pattern ( **Fig 1G and H** ). The punctate pattern was observed in 50% (18/36) of spermatozoa, and PA5-59184 labeling colocalizes discretely with the centrin-labeled proximal and distal centrioles ( **Fig 1C-D** ). The antibody colocalization with centrin labeling was higher in the proximal centriole than the distal centriole (PC=0.47±0.31, DC=0.40±0.34, P=0.05, N=33, paired t-test). The punctate pattern had a moderate positive colocalization between the PA5-59184 labeling and centrin at the proximal centriole (0.47) and the distal centriole (0.40) using the Fisher’s z transformation method (Sanchez-Meca et al., 2013). In contrast, the dispersed pattern has diffuse staining over the region of the spermatozoon neck. The dispersed staining included both the centrin-labeled proximal and distal centrioles and their surroundings and was also evident in half of the spermatozoa (18/36).

PA5-59184 labeling was more intense at the distal centriole than the proximal centriole in the punctate phenotype (47%, 1.47±0.32-fold, P=0.07, N=18, paired t-test) and the dispersed phenotype (40%, 1.40±0.44-fold, P=0.004, N=18, paired t-test). A similar labeling difference is observed in other distal centriole proteins, such as POC1B and FAM161A, but not centrin (Fishman et al., 2018; Khanal et al., 2021) . As expected, the punctate pattern labeling was more intense in both the proximal and distal centrioles than in the axoneme (proximal centriole compared to axoneme, N=18, 17±4, P=0.0002, t-test; distal centriole compared to axoneme, N=18,17±4, P=3E-9, paired t-test). Similarly, the dispersed pattern labeling was more intense in both the proximal and distal centrioles than in the axoneme (proximal centriole compared to axoneme, N=18, 13±6, P=0.02, paired t-test; distal centriole compared to axoneme, N=18, 13±6, P=0.0001, paired t-test). Since the axoneme is not expected to have CCDC15, the axoneme serves as a background control, suggesting that dispersed patterns in the neck represent a true labeling pattern rather than nonspecific background labeling. Also, both patterns were observed in samples from three distinct men (sample 1: thirteen disperse versus one punctate, sample 2: three disperse versus eight punctate, sample 3: two disperse versus nine punctate), suggesting they are not due to specific differences in genetic background or environmental factors.

The second CCDC15 antibody found similar results to the first one, validating the findings. The second commercial CCDC15 antibody was a rabbit polyclonal antibody raised against the first 951 amino acids of human CCDC15 (HPA041738). We found the punctate phenotype in 40% (14/35) and the dispersed phenotype in 60% (21/35) of spermatozoa. HPA041738 colocalization with centrin labeling was higher in the proximal centriole compared to the distal centriole (PC=0.44±0.18, DC=0.28±0.27, P=2E-5, N=35, paired t-test). HPA041738 labeling intensity was 15% more intense at the distal centriole than the proximal centriole in the punctate phenotype (1.15±0.27-fold, P=0.25, N=14, unpaired t-test) and 29% more intense at the proximal centriole than the distal centriole in the dispersed phenotype (1.29±0.40-fold, P=0.9, N=21, unpaired t-test). The punctate pattern labeling of HPA04173 was more intense in both the proximal and distal centrioles than in the axoneme (proximal centriole compared to axoneme, N=14, 2±1.6, P=2E-4, paired t-test; distal centriole compared to axoneme, N=14,2±1.6, P=3E-4, paired t-test). HPA041738 labeling intensity was 15% more intense at the distal centriole than the proximal centriole in the punctate phenotype (1.15±0.27-fold, P=0.25, N=14, unpaired t-test) and 29% more intense at the proximal centriole than the distal centriole in the dispersed phenotype (1.29±0.40-fold, P=0.9, N=21, unpaired t-test). The punctate pattern labeling of HPA04173 was more intense in both the proximal and distal centrioles than in the axoneme (proximal centriole compared to axoneme, N=14, 2±1.6, P=2E-4, paired t-test; distal centriole compared to axoneme, N=14,2±1.6, P=3E-4, paired t-test). The dispersed pattern labeling of HPA04173 was also more intense in both the proximal and distal centrioles than in the axoneme (proximal centriole compared to axoneme, N=21, 1.5±1.1, P=4E-5, paired t-test; distal centriole compared to axoneme, N=21, 1.5±1.1, P=3E-5, paired t-test). Again, both patterns were observed in significant proportions (Sample 1: four disperse versus six punctate, N=10; Sample 2: ten disperse versus three punctate, N=13; Sample 3: seven disperse versus five punctate, N=12).

Centrosomal protein of 44 KDa (CEP44) is a centriole-specific protein that localizes near the mother and daughter centrioles’ base (like CEP135) in somatic cells (Hossain et al., 2020) . It is essential for proper centriole structure and recruitment of CEP135 to the daughter centriole while partially co-localizing with POC1B in somatic cells (Atorino et al., 2020) . Additionally, CEP44 is involved in recruiting and stabilizing centrosome linker proteins, a proteinaceous structure connecting somatic centrioles during interphase (Hossain et al., 2020) . Stimulated emission depletion (STED) imaging of HeLa cells shows CEP44 localizes to the base of microtubule triplets, forming a ~190nm ring of nine organized signals within the centriolar inner scaffolding (Sullenberger et al., 2023) . Its localization in spermatozoa is unknown.

To study CEP44 localization in the sperm centriole, we used two commercial antibodies that recognize it: 24457-1-AP (Proteintech) and HPA041835 (Sigma) using methanol fixatives. These antibodies did not consistently label the sperm neck using formaldehyde as a fixative.

24457-1-AP is a rabbit polyclonal antibody recognizing CEP44 amino acids 1 to 350. This antibody has been used successfully to determine the localization of CEP44 within centrioles of somatic cells (Hossain et al., 2020) . Like anti-CEP135 labeling, we found that 24457-1-AP labeled both the proximal and distal centrioles ( **Fig 1I and J).** The labeling was more intense at the proximal centriole (1.61±0.65-fold, P=3E-9, N=36, paired t-test) than at the distal centriole (Turner, Caswell, et al., 2023) . However, the antibody colocalization with centrin labeling was similar in both the proximal and distal centriole (PC=0.31±0.29, DC=0.40±0.23, P=0.08, N=36, paired t-test).

The anti-CCDC15 antibody PA5-59184 had a 58% greater colocalization with centrin in the proximal centriole compared to the anti-CEP44 antibody 24457-1-AP (1.58±1, P=0.02, N=64, t-test), but had similar colocalization with centrin in the distal centriole compared to the anti-CEP44 antibody 24457-1-AP (1.00±0.7, P=0.99, N=64, t-test).

The second commercial CEP44 antibody, HPA041835, recognizes CEP44 amino acids 216 to 294. We found that HPA041835 demonstrated a similar localization pattern to that of 24457-1-AP, consistently labeling near the base of both the proximal and distal centriole with similar colocalization to centrin in both the proximal and distal centriole ( **Fig 1K and L)** (PC=0.24±0.20, DC=0.25±0.14, P=0.7, N=33, paired t-test). Like 24457-1-AP, HPA041835 labeled the proximal centriole more intensely than the distal centriole (1.43±0.57-fold, P=0.0004, N=33, paired t-test).

The anti-CCDC15 antibody HPA041738 had a 94% greater colocalization with centrin in the proximal centriole compared to the anti-CEP44 antibody HPA041835 (1.94±0.92, P=1E-5, N=67, unpaired t-test). However, no difference was observed in colocalization with centrin labeling in the distal centriole between anti-CEP44 antibody 24457-1-AP and anti-CCDC15 antibody PA5-59184 (1.12±1.95, P=0.57, N=67, unpaired t-test).

No specific labeling was observed when spermatozoa were stained without the anti-CCDC15 and anti-CEP44 antibodies but with secondary antibodies (Extended Data).

Overall, our studies in human spermatozoa found that two independently raised CCDC15 antibodies had similar labeling to centrin, suggesting CCDC15 and centrin have a related function, possibly at the proximal centriole distal lumen and distal centriole rods. CCDC15 has two distinct labeling patterns that are very different, raising questions about what is underlying these differences. These dichotomic patterns are unique to this protein and were not reported in the ten or more sperm centriole proteins studied in the past. Also, we studied two independently raised CEP44 antibodies that had similar labeling near centrin, a similar localization we reported for CEP135 (Turner, Caswell, et al., 2023) . This suggests that CEP44 and CEP135 may have a related function at the proximal and distal centriole bases. Together, this study demonstrates that CCDC15 and CEP44 are components of the human spermatozoa, possibly using them as a biomarker in evaluating sperm centriole quality in methods such as Fluorescence-Based Ratiometric Analysis of Sperm Centrioles (FRAC) (Jaiswal et al., 2022; Turner, Achinger, et al., 2023; Turner et al., 2021) .

## Methods

Sperm preparation : The slides were placed in a Coplin jar with methanol that was pre-chilled to -20 °C for three minutes (CEP44) or in a Coplin jar with room temperature formaldehyde for ten minutes (CCDC15) to ensure proper fixation of sperm. Room temperature PBS was then used to wash the slides directly following fixation. Slides were then transferred to PBS with 0.3% Triton x-100 (PBST) for 60 minutes to permeabilize the sample, followed by a blocking step in PBST with 1% BSA w/v (PBSTB) for 30 minutes. A primary antibody solution was prepared in PBSTB and then added to the slides. The slides were then covered with parafilm and incubated for 17 hours in a humidity chamber at 4 °C. The slides were then washed in PBST three times for five minutes each. The secondary antibodies were prepared in PBSTB and added to the slides, followed by an incubation period of four hours in a humidity chamber at room temperature. The slides were washed in PBST three times for five minutes each, followed by three additional washes in PBS for five minutes each. A single drop of Fluoroshield with DAPI was placed onto the slides, followed by the addition of a cover slip. The slides were then sealed with nail polish and stored in a fridge at 4 °C until imaging.

Slide preparations: a 50 µL droplet of sperm from suspension was pipetted onto each slide, and a coverslip was placed on the respective droplet. Snap-freezing was accomplished by submerging the slide into liquid nitrogen following coverslip placement. A liquid nitrogen tank was then used to store slides until they were used for staining.

Sperm visualization: Slides were visualized using a Leica SP8 confocal microscope in BrightR mode using an HC PL APO CS2 63x/1.40 OIL lens, 100% gain, 512x512 pixels (155µM x 155µM) format, 6x zoom factor, maximum line averaging of 1, frame accumulation of 1, and occasional rotation. Four sequences were used to collect the fluorescence signals. DNA and phase-like images were produced in the first sequence using a 410 nm laser, and the emissions were captured using the HyD1 detector to detect photons between 410-478 maximum and were color-coded to blue. The fluoro-turret was set to Scan-PH to get a phase-like image, and PMT Trans was set to ON with a gain of 250 in greyscale. CEP44 or CCDC15 and Centrin images were collected in the second sequence, with the anti-rabbit secondary conjugated to ALEXA 488, and were activated using a 488 nm laser, with the absorption spectrum set to 511-564 nm. Centrin, which is also in the second sequence, was acquired by activating the anti-mouse secondary, which was conjugated to ALEXA 647 with a 633 nm laser, and the absorption spectrum was set to 721-774nm via the HyD4 detector and was color-coded to magenta. Tubulin images were captured in the third sequence, and the anti-sheep secondary was conjugated to ALEXA 555, which a 561 nm laser was set to. The absorption spectrum was set to 566-623 nm via the HyD3 detector and was color-coded to red. We collected between 10-20 Z sections of 0.3 µM thickness from the top to the bottom of the sperm. At least three independent staining were performed with similar results.

Colocalization: Using the LasX colocalization feature, the parameters of the threshold for both anti-centrin and anti-CEP44/CCDC15 channels at 30% and background for both anti-centrin and anti-CEP44/CCDC15 channels at 20% were established. A region of interest (ROI) was created at 0.5 µm width and 0.75 µm height. This ROI was centered around the proximal centriole, and the corresponding data was copied into Excel. The same process was repeated for the distal centriole. Pearson’s correlation value was converted into a Fisher’s z value in Excel (Sanchez-Meca et al., 2013). This was repeated for all Pearson’s correlation values obtained for both the proximal and distal centrioles. The average of these Fisher’s z values was obtained, and then the Fisher inverse function was utilized on the average value. This was done for each sample, as well as overall between all the samples.

Intensity: We used the LasX intensity feature. A region of interest (ROI) was created at 0.5 µm width and 0.75 µm height. The ROI was centered around the proximal centriole, and the corresponding data was copied into Excel. This process was repeated for the distal centriole and the axoneme. The axoneme ROI was established at 2 µm from the distal centriole. The average intensity of centrin in the proximal centriole and the distal centriole was obtained, as well as the standard deviation. The average intensity of CEP44 and CCDC15 was also obtained for both the proximal and distal centrioles. This was done using the mean value acquired from LasX.

## Reagents

*Primary Antibodies:*

Anti-CEP44 was made in rabbits (Proteintech, 24457-1-AP). This rabbit polyclonal antibody was raised against the first 350 amino acids of human CEP44 (Diluted 1/35).

Anti-CEP44 was made in rabbits (Sigma-Aldrich, HPA041835). This rabbit polyclonal antibody recognizes CEP44 amino acids 216 to 294 (Diluted 1/50).

Anti-CCDC15 was made in rabbits (Thermo Fisher Scientific, PA5-59184). This rabbit polyclonal antibody was raised against amino acids 291-370 against human CCDC15 (Diluted 1/15).

Anti-CCDC15 was made in rabbits (Sigma-Aldrich, HPA041738). This rabbit polyclonal antibody was raised against the first 951 amino acids of human CCDC15 (Diluted 1/100).

Anti-centrin made in Mouse (Sigma-Aldrich, 04-1624) (Diluted 1/70).

Anti-tubulin made in Sheep (Cytoskeleton, ATN02) (Diluted 1/600).

*Secondary Antibodies:*

Anti-Mouse DyLight 488 (ThermoFisher Scientific, A-21436) (Diluted 1/400).

Anti-Sheep Alexa 555 (ThermoFisher Scientific, SA5-10166) (Diluted 1/1000).

Anti-Rabbit Alexa 650 (ThermoFisher Scientific, SA5-10041) (Diluted 1/400).

Fluoroshield containing DAPI (Sigma-Aldrich, F6057-20ML) (1 drop).

Solutions:

Washing solution: PBS

Permeabilization Buffer (PBST): is made of PBS with 0.3% Triton X-100 (Sigma Aldrich, 9002-93-1)

Blocking Solution (PBSTB): Is made of PBST with 1% BSA (Bovine Serum Albumin) (CHEM-IMPEX INT’L, 00535)

Materials:

Slides and Cover Slips: Glass slide (Azer Scientific, EMS200A+), glass coverslip (VWR, 48366-205)

Glass Coplin jars: Research Products International Corp. Cat. # 50-212-281

We used presumed fertile men's sperm from the Reproductive Subject Registry and Sample Repository (RSRSR) (Schon et al., 2021) at the University of Michigan (UM IRB#HUM00125627). The Institutional Review Board (IRB) at the University of Toledo approved this study (UT IRB#300364; initial approval 10/11/2019; PI: Tomer Avidor-Reiss).
